# Weak-to-strong generalization enables fully automated training of multi-head mask-RCNN model for segmenting densely overlapping cell nuclei in multiplex whole-slice brain images

**DOI:** 10.3389/fbinf.2026.1733655

**Published:** 2026-05-11

**Authors:** Lin Bai, Xiaoyang Li, Liqiang Huang, Quynh Nguyen, Hien Van Nguyen, Saurabh Prasad, Dragan Maric, John Redell, Pramod Dash, Badrinath Roysam

**Affiliations:** 1 Department of Electrical and Computer Engineering, University of Houston, Houston, TX, United States; 2 Canva Pty Ltd., Sydney, NSW, Australia; 3 National Institute of Neurological Disorders and Stroke, Bethesda, MD, United States; 4 Department of Neurobiology and Anatomy, The University of Texas McGovern Medical School, Houston, TX, United States

**Keywords:** automated training, cell nuclei, instance segmentation, multiplex immunofluorescence, weak to strong learning, whole-slide imaging

## Abstract

We present a weak to strong generalization methodology for fully automated training of a multi-head extension of the Mask-RCNN method with efficient channel attention for reliable segmentation of overlapping cell nuclei in multiplex cyclic immunofluorescent (IF) whole-slide images (WSIs), and present evidence for pseudo-label correction and coverage expansion, the key phenomena underlying weak to strong generalization. This method is designed to enable domain adaptation for multiplex spatial proteomics imaging data, eliminating the need for additional human annotations in the target domain. We also present metrics for automated self-diagnosis of segmentation quality in production environments, where human visual proofreading of massive WSI images is unaffordable. Our method was benchmarked against five current widely used methods and showed a significant improvement. The code, sample WSI images, and high-resolution segmentation results are provided in open form for community adoption and adaptation.

## 1 Introduction

Multiplex immunofluorescent (IF) imaging of whole slides of tissues using automated cyclic staining is currently performed on a large scale for deep cellular characterization of histological samples in pre-clinical science and drug discovery (Al-Kofahi et al., 2009; Li et al., 2017; Li et al., 2022; Lin et al., 2003; Lin et al., 2007; Lin et al., 2005; Maric et al., 2021; Rivest et al., 2023). These systems generate massive multi-gigabyte multi-channel whole-slide images (WSIs) containing hundreds of thousands or more cells that must be analyzed accurately with maximum-possible and scalable automation. Reliable detection and accurate segmentation of cell nuclei is a critical yet challenging first step towards cell-based quantification of molecular markers. In spatially resolved tissue analysis, the accurate computational segmentation of individual cells is of paramount importance. Unlike dissociative methods, *in situ* imaging requires the delineation of cells from densely packed and often overlapping neighbors. Errors in this critical step directly propagate to downstream analyses: imprecise boundaries cause signal spillover that distorts molecular expression profiles, leading to erroneous cell type classification and biased spatial quantification. On the scale of a whole-slide image, containing millions of cells, even minor segmentation inaccuracies result in substantial cumulative errors, undermining analytical integrity and necessitating extensive manual proofreading (Rumberger et al., 2025). This problem is especially challenging since WSI images cover extended tissue regions that can exhibit high variability in staining, tissue architecture, and cell morphology, and varied image artifacts arising from automated scanning and cyclic immunofluorescence processes. Interestingly, even the fluorescent staining of cell nuclei is highly variable across WSI tissue regions and the commonly used DNA stain DAPI does not mark all nuclei as evident in Figure 1A showing an image of a rat brain slice (29,398 × 43,054 pixels per channel, >250,000 cells) drawn from a 50-plex dataset (Maric et al., 2021) (The full high-resolution image is posted in the Supplement).

**FIGURE 1:**
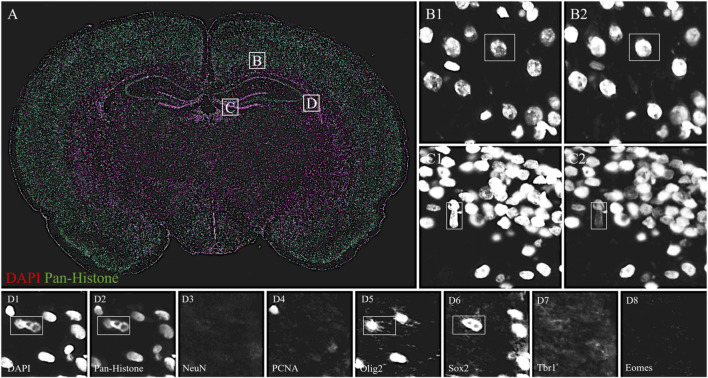
Sample 8-plex image of a whole rat brain slice 29,398 × 43,054 pixels per channel, >250,000 cells) showing major spatial variations in DAPI and histone labeling. **(A)** RGB rendering with DAPI in Red, Pan-Histone in Green, and their average in blue. **(B1, B2)** Region B showing strong histone labeling but weak DAPI labeling. **(C1, C2)** Region C showing strong DAPI labeling but weak histone labeling. **(D1–D8)** Region D in which we see a cluster of 3 cells that are difficult to separate based on nuclear stains alone that become possible to distinguish when cell-type channels Olig2 and Sox2 are utilized.

Recent progress in segmenting cell nuclei has largely been driven by the application of deep neural networks and foundational models trained on massive human-annotated datasets (Kirillov et al., 2023; Radford et al., 2021; Ramesh et al., 2022) in a bid to capture the variability. Despite the progress, these methods remain human annotation effort intensive. Foundational models require massive initial annotation efforts to train, and additional annotation efforts to adapt the models to a new class of images. Even then, several problems remain. For example, the correct handling of overlapping nuclei in thicker samples remains unaddressed (Figure 2). Next, foundational models are often trained on 3-channel (RGB) images and therefore cannot exploit the cues that are available in multiplex WSI images, a missed opportunity (Figure 1). Finally, the problem of assessing the accuracy of automatically generated segmentations remains human effort intensive. There is a compelling need for automated assessment methods, especially in production environments, where visual proofreading of massive WSI images is unaffordable and not scalable.

**FIGURE 2:**
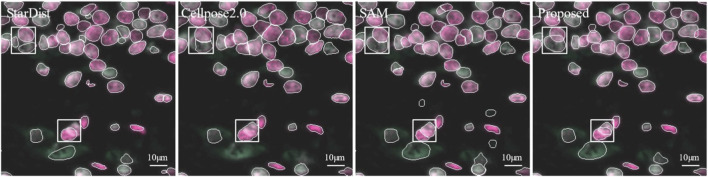
Foundation models (StarDist, Cellpose, and SAM) exhibit poor performance in segmenting overlapping/clustered nuclei. For example, they either over segment (StarDist) or under segment (Cellpose) or only able to segment the clearly visible part of nuclei body (SAM). However, these methods can be used to generate automated weak annotations to train our strong model that produces accurate results without requiring any human annotation effort.

We present an integrated *fully automated* methodology based on *weak-to-strong generalization* (Lang et al., 2024) to overcome the above-mentioned limitations (Figure 3), and metrics for automated segmentation quality assessment. Weak-to-strong generalization is a special case of weakly supervised machine learning in which a strong model (student) is trained using (comparatively unreliable) annotations generated automatically by a weaker model (teacher) after they are subjected to well-designed automated cleanup and data augmentation steps. This strong model is trained using weak annotations generated using a current foundational model (e.g., the Segment Anything Model, Kirillov et al., 2023) that lacks needed capabilities like the ability to handle overlapping nuclei. In this work, we refer to these weak annotations as “pseudo labels” in line with the weak-to-strong generalization literature. A key step is the automated processing of the pseudo labels to generate a large collection of synthetic examples with numerous instances of complex and variable nuclear overlap patterns. From these augmented weak label data, we train the strong model by weak-to-strong learning (Lang et al., 2024). Lang et al. (2024) showed that a well-trained student model can learn to correct the weaker model’s mistakes (“pseudo label correction”) and generalize to instances where the teacher lacks confidence, even when these examples are excluded from the training data (“coverage expansion”). We present evidence for these phenomena and show their beneficial impact. Finally, we present metrics for assessing the accuracy of large-scale nuclear segmentations in a fully automated manner based on accounting for the fluorescence signal (coverage) and quantifying the purity of fluorescent signatures over cells.

**FIGURE 3:**
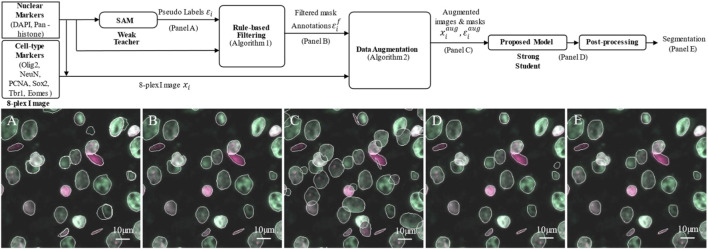
Overview of the weak-to-strong learning framework. The foundation model SAM acts as a weak teacher to generate annotations, which are used to train our model as a strong student. **(A)** DAPI and Pan-Histone images with automated point prompts are input to SAM to generate mask annotations. **(B)** A rule-based filtering step removes union and duplicate masks to ensure annotation quality. **(C)** The filtered masks and 8-plex images undergo instance-level data augmentation. **(D)** The augmented data is used to train the proposed model. **(E)** Post-processing produces the final segmentation results.

## 2 Summary of prior work

Methods for segmenting cell nuclei fall into three main categories. First, there are parameter-based methods, such as thresholding (Otsu, 1979), watershed (Malpica et al., 1997), or active contours (Chan and Vese, 2001), that require users to manually adjust parameters for each imaging modality and scale. This requires care and expertise and often yields suboptimal performance, largely due to variability across the large WSI images. The second involves deep CNN models (e.g., Mask-RCNN (He et al., 2017), U-Net (Ronneberger et al., 2015), YOLACT (Bolya et al., 2019)) that are custom trained for each class of images to cope with the expected variability. Variations of these models (Graham et al., 2019; Jia et al., 2021; Johnson, 2018; 2020; Long, 2020; Lv et al., 2019; Vuola et al., 2019; Zhou et al., 2018) have been extensively applied to nuclear segmentation. Deep Learning Models (Ke et al., 2021; Kong et al., 2020; Molnar et al., 2016; Roy et al., 2025; Zhou et al., 2019) like DoNet (Jiang et al., 2023) are specifically designed to identify overlapping objects, and many augmentation methods (Dvornik et al., 2018; Dwibedi et al., 2017; Fang et al., 2019; Ghiasi et al., 2021; Lin et al., 2022; Yu et al., 2023; Yun et al., 2019; Zhang, 2017) are proposed to boost segmentation performance. While these methods achieve high accuracy, they require extensive annotation efforts, making them time-consuming and resource intensive. The third and most scalable approach is to develop reusable deep learning based models like StarDist (Schmidt et al., 2018), Cellpose (Pachitariu and Stringer, 2022; Stringer and Pachitariu, 2025; Stringer et al., 2021), CellSeg (Lee et al., 2022) that are pre-trained on large and diverse datasets. While these models are efficient and widely used, they often suffer from domain shift–when the test images arise from a distribution that is different from the training set, and the model’s performance can degrade. Furthermore, these methods are designed to work with a small number of channels (e.g., RGB images), making them sub-optimal in multiplex WSI applications.

Recently, large-scale, pre-trained foundation models (e.g., the Segment Anything Model (SAM) (Kirillov et al., 2023)) trained on >1B masks over 11M images show competitive segmentation performance and often outperform fully-supervised models. Recent work has also explored adapting SAM for biomedical image segmentation through fine-tuning or prompt engineering strategies (e.g., All-in-SAM (Cui et al., 2024), MedSAM (Ma et al., 2024), and μSAM (Archit et al., 2025). These approaches improve SAM’s performance in domain-specific settings but typically rely on additional annotated data for adaptation. In parallel, several occlusion-aware instance segmentation methods have been proposed to explicitly model overlapping objects, particularly in microscopy images. While these approaches provide valuable insights for handling occlusions, they generally assume access to high-quality annotations during training. In contrast, our approach focuses on a fully automated weak-to-strong learning pipeline that leverages SAM-generated pseudo-labels and automated cleanup to train a stronger segmentation model without requiring additional human annotations. Foundation models can generalize data distributions beyond those encountered during training, a capability facilitated by prompt engineering and referred to as zero-shot and few-shot generalization. Although powerful, models like SAM cannot handle overlapping nuclei, and multiplex images since they are trained on RGB images, and require manual prompts to generate segmentations, limiting their applicability in fully automated workflows. Many methods to fine tune SAM for biomedical images have been described, such as All-in-SAM (Cui et al., 2024), fine-tuning SAM by providing box level annotations, μSAM (Archit et al., 2025), fine-tuning SAM for light and electron microscopy, MedSAM (Ma et al., 2024), fine-tuning SAM on an unprecedented dataset with more than one million medical image-mask pairs. Unfortunately, fine-tuning SAM requires a large corpus of (expensive) domain-specific annotation data.

In this paper we show how we can leverage existing models (e.g., SAM) as a teacher model to generate pseudo labels for training a more capable student model. In weakly supervised learning, for instance, co-teaching (Han et al., 2018), involves training two network to teach the other. Co-teaching+ (Yu et al., 2019) builds on this strategy by selecting low-loss points from samples where the two networks disagree. Co-learning (Tan et al., 2021) integrates supervised and self-supervised learning with a shared feature encoder with two exclusive heads, that provide different views of the data. JoCoR (Zhang et al., 2018) aims to reduce the diversity of two networks during training. Compared to classification, fewer studies focus on segmentation tasks. ADELE (Liu et al., 2022) employs an early stopping strategy to allow the network to fit clean labels before it memorizes false annotations. Recently, some authors have developed theoretic support for weak-to-strong learning (Lang et al., 2024) for classification tasks, and identified the conditions under which this strategy is successful. Our work aims to leverage these emerging insights and adapt weak to strong learning to instance segmentation.

## 3 Weak to strong learning methods

Figure 3 depicts the main steps of the proposed weak-to-strong learning approach. For clarity, we summarize the key components of the pipeline here, while detailed pseudocode and implementation steps are provided in the Supplementary Appendix A1-3. For simplicity, we illustrate the training procedure using a single multiplex image III; the same procedure naturally extends to a batch of images. The trained model can then be used to segment similar images in inference mode.

Throughout this section, $x_{i}$ denotes an image tile, $\varepsilon_{i}$ denotes the corresponding set of pseudo-label masks generated by SAM, and $\varepsilon_{i,j}$ denotes the $j^{th}$ mask in the $i^{th}$ tile.

### 3.1 Automated pseudo-label generation using the Segment Anything Model

Given a large multiplex WSI image I , we dice it into a set of K tiles ${\left\{x_{i}\right\}}_{i=1}^{K},$ each of size 512 × 512 pixels. We extract a second set of two-channel tiles ${\left\{x_{i}^{DAPI,Histone}\right\}}_{i=1}^{K}$ composed of the nuclear markers DAPI and Pan-histone. From this set, we construct a dataset $D={\left\{\left(x_{i}^{DAPI,Histone},\varepsilon_{i}\right)\right\}}_{i=1}^{K}$ where $\varepsilon_{i}={\left\{\varepsilon_{i,j}\right\}}_{j=1}^{N_{i}}$ is the set of masks (pseudo labels) generated by SAM using an array of point prompts, $N_{i}$ is the number of masks in the $i^{th}$ tile. In our implementation, these point prompts are generated automatically as a regular grid over each tile (Figure 3A). For SAM-C, we use a 256 × 256 array of point prompts as the default setting. Given the masks $\varepsilon_{i}$ may include noisy labels due to false detections, over segmentations, under segmentations, or erroneous overlaps, we introduce a simple rule-based strategy to filter out these errors without human-efforts, which include union masks (two or more smaller masks that are subsumed by a larger mask), and duplicate masks. The output of the rule-based filtering is denoted $D^{f}={\left\{\left(x_{i}^{DAPI,Histone},\varepsilon_{i}^{f}\right)\right\}}_{i=1}^{K}$ , where $\varepsilon_{i}^{f}={\left\{\varepsilon_{i,j}^{f}\right\}}_{j=1}^{N_{i}^{f}}$ , and $N_{i}^{f}$ indicates the revised count of instances in the $i^{th}$ image. Subsequently, to simulate cell overlap scenarios during training, we apply a simple copy–paste augmentation strategy that overlays transformed nuclei onto other nuclei within the same tile to generate augmented samples. The details of augmentation is provided in Supplementary Appendix A3.

### 3.2 The proposed strong model (M^3^-RCNN)

Figure 4 depicts the architecture of the proposed strong M^3^-RCNN model. We use the Mask-RCNN (He et al., 2017) as the starting point given its competitive performance in instance segmentation. It operates in two stages. The first stage employs a backbone network like ResNet-50 (He et al., 2016) and a feature pyramid network (FPN) to extract multiscale features of each image tile $x^{i}$ . A region proposal network (RPN) identifies boxed regions of interest (ROI), and their features $f_{i,j}^{ROI}$ . The second stage consists of a bounding box regression head, classification head and a mask head that uses the ROI features $f_{i,j}^{ROI}$ to generate bounding boxes, object classifications and segmentation masks. The composite loss function of Mask-RCNN is articulated as shown in Equation 1:

$$L_{MRCNN\;}={\lambda_{1}L}_{reg}+{\lambda_{2}L}_{cls}+\lambda_{3}L_{mask,}$$ (1)

where $L_{reg}$ represents the smooth-L1 loss for bounding box regression in two stages, $L_{cls}$ is the cross-entropy loss for classification in two stages, $L_{mask}$ is the pixel-wise cross-entropy loss for segmentation in stage two, and $\lambda_{1},\lambda_{2}$ , $\lambda_{3}$ are loss weights. Here, given that we are analyzing eight-channel images, we use an Efficient Channel Attention (ECA) module (Wang et al., 2020) to weight each channel’s feature map to compute the overall feature map $f_{i,j}^{ECA}$ for each box. The ECA module consists of three components, global average pooling (GAP), one-dimensional convolution ( W ), and a sigmoid function σ . The input to the ECM is $f_{i,j}^{ROI}$ for the $j^{th}$ object from $i^{th}$ image tile, and the output $f_{i,j}^{ECA}$ is given by Equation 2:

$$f_{i,j}^{ECA}=\sigma \left(W*GAP\left(f_{i,j}^{ROI}\right)\right)$$ (2)

**FIGURE 4:**
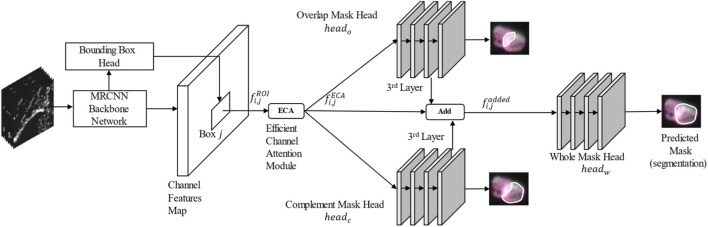
Overview of the proposed M3-RCNN architecture. DAPI and Pan-Histone channels are used for nucleus detection to generate bounding boxes, while all eight channels are used for segmentation. Features within each detected region are processed by an Efficient Channel Attention (ECA) module to weight channel contributions, followed by convolutional layers for feature refinement. The refined features are fed into three heads—Overlap, Complement, and Whole Mask—to predict different components of the final segmentation. The outputs of the Overlap and Complement heads are further integrated to guide Whole Mask prediction.

To leverage these weak but important cues, we diverge from the conventional single-mask-head framework and introduce two additional mask heads specifically designed to capture the overlapping and complement parts of the objects, alongside the standard whole object representation, as illustrated in Figure 4. We denote these mask heads $head_{c}$ , $head_{w},head_{o,}$ for the complement, whole mask, and overlap mask heads, respectively. As shown in Figure 4, the inputs of $head_{c}$ and $head_{o}$ are $f_{i,j}^{ECA}$ , and the output feature maps of the $head_{c}$ and $head_{o}$ from the 3^rd^ convolution layer are added with $f_{i,j}^{ECA}$ to obtain the feature map $f_{i,j}^{added}$ of the whole mask head. The loss functions for the mask heads are formulated as shown in Equation 3. The full formulation is provided for completeness, while the main intuition is that these heads separately model overlapping, complementary, and whole-object regions. Specifically, the loss for the whole mask head is defined in Equation 4, while the overlap and complement losses are defined in Equations 5, 6, respectively.

$$L_{mask}^{nonconcave}=\frac{1}{k}\sum_{i=1}^{k}\frac{1}{N_{i}^{aug,nc\;}}\sum_{j=1}^{N_{i}^{aug,nc\;}}(L_{w}+L_{c}+L_{0});$$ (3)

$$L_{w}{=L}_{ce}\left(head_{w}\left(f_{i,j}^{added}\right),e_{i,j}^{aug}\right);$$ (4)

$$L_{o}{=L}_{ce}\left({head}_{o}(f_{ECA}^{i,j}),e_{i,j}^{aug,o}\right);$$ (5)

$$L_{c}{=L}_{ce}\left({head}_{c}(f_{ECA}^{i,j}),e_{i,j}^{aug,c}\right);$$ (6)

where $L_{mask}^{non-concave}$ is the loss for non-concave samples and $N_{i}^{aug,nc\;}$ represents the total number of instances with non-concave contours, $e_{i,j}^{aug,o}$ and $e_{i,j}^{aug,c}$ represent the overlapping and complement parts of the augmented mask $e_{i,j}^{aug}$ , respectively, and $L_{ce}$ is the cross-entropy loss function. Given two overlapping nuclei A and B, the segmentation from SAM generally yield a comprehensive mask for nucleus A and a complementary mask for nucleus B. When analyzing its segmentation output, denoted as $e_{i,j}^{aug}$ and armed with the prior understanding that the typical shape of nuclei is non-concave, we categorize these masks into concave (caused by overlapping) and non-concave based on the morphology of their contours. Masks exhibiting non-concave outlines are considered accurate and can be accepted as is. Masks with concave or incomplete outlines are excluded from the mask loss computation due to their structural irregularity. However, these instances are still utilized in the classification and bounding box regression losses, as their bounding boxes can remain accurate when overlap is not severe, despite the mask being incomplete. Our M^3^-RCNN model is trained in an end-to-end manner using a composite loss function as defined in Equation 7:

$$L_{M3RCNN\;}={\lambda_{1}L}_{reg}+\lambda_{2}L_{cls}+\lambda_{3}L_{mask}^{non-concave}$$ (7)

## 4 Experimental results

The materials and methods for the multiplex WSI imaging are described in our earlier paper and the data are publicly available (Maric et al., 2021). We evaluated the proposed method against five current methods (Table 1; Table 2) in two different ways: (i) automated (unsupervised) assessment using novel metrics described below; and (ii) manual (supervised) assessment against ground truth annotations. The natural near symmetry of the rat brain provides an efficient and practical way to perform these steps. First, our proposed M^3^-RCNN model was trained on K=2,494 image tiles of size 512 × 512 pixels cropped from the left side of the first whole rat brain slice image (S1). The unsupervised assessment was carried out over all the brain slices (S1, … S4), by dividing the WSI images into tiles of size 512 × 512 pixels with 50 pixels overlap. Unless otherwise stated, we use the same tiling scheme for pseudo-label generation and evaluation, and the overlap controls the effective stride in whole-slide inference. For the more effort-intensive supervised assessment, we randomly selected 100 tiles from the right side of the brain in the S1 dataset, annotated them using the semi-automated online computer vision annotation tool (CVAT), and used this as the ground truth dataset.

**TABLE 1:** Performance metrics on the S1 dataset.

Methods	Channels	S1				
		AJI+	PQ	Coverage	Purity	Cell counts
*Cellpose2.0*	1-plex	65.6%	65.2%	82.89%	98.99%	193,658
*StarDist*	1-plex	67.4%	68.1%	88.47%	99.19%	247,103
*SAM-C*	2-plex	76.0%	74.7%	97.62%	99.12%	257,860
*MRCNN*	2-plex	76.3%	76.0%	98.04%	**99.30%**	**260,267**
*MRCNN-AUG*	2-plex	76.2%	75.9%	**98.26%**	99.17%	258,782
*M3-RCNN*	8-plex	**77.3%**	**76.7%**	98.22%	99.19%	255,726

Bold values indicate the best performance for each evaluation metrics.

**TABLE 2:** Performance metrics on the S2, S3, S4 datasets.

Methods	Channels	S2			S3			S4		
		Coverage	Purity	Cell counts	Coverage	Purity	Cell counts	Coverage	Purity	Cell counts
*Cellpose2.0*	1-plex	74.56%	98.57%	175,823	84.11%	99.11%	197,136	81.17%	98.60%	193,264
*StarDist*	1-plex	93.81%	98.94%	**250,311**	88.66%	99.40%	240,768	88.11%	99.11%	243,169
*SAM-C*	2-plex	91.88%	98.88%	248,263	97.65%	99.33%	**266,559**	97.40%	99.02%	**272,346**
*MRCNN*	2-plex	93.12%	**99.00%**	237,956	99.05%	**99.40%**	252,179	98.57%	**99.11%**	256,516
*MRCNN-AUG*	2-plex	**94.26%**	98.93%	243,830	**99.44%**	99.36%	257,147	**99.00%**	99.03%	260,994
*M3-RCNN*	8-plex	93.93%	98.90%	241,796	98.39%	99.39%	255,823	98.61%	99.01%	256,789

1-plex: DAPI, 2-plex; DAPI, Pan-histone, 8-plex: DAPI, Pan-histone, NeuN, Olig2, PCNA, Sox2, Tbr1, Eomes.

SAM-C: SAM-Cleaned (Teacher), MRCNN-AUG: Mask-RCNN + Augmentation.

Bold values indicate the best performance for each evaluation metrics.

Finally, to show the pseudo-label correction phenomenon under our weak-to-strong generalization framework, we randomly selected 100 tiles from our training set (left side of the S1 brain), annotated them using the CVAT annotation tool, and visualized this phenomenon using t-SNE multi-dimensional projection (Van der Maaten and Hinton, 2008).

For supervised evaluation, we use Aggregated Jaccard Index plus (AJI+) (Graham et al., 2019) and Panoptic Quality (PQ) (Kirillov et al., 2019) as our primary metrics. AJI + improves upon the original Aggregated Jaccard Index (AJI) (Kumar et al., 2017) by penalizing unmatched ground truth instances. PQ jointly evaluate detection and segmentation quality in a single metric. Before introducing the quantitative metrics, we first illustrate qualitative segmentation results in Figure 5.

**FIGURE 5:**
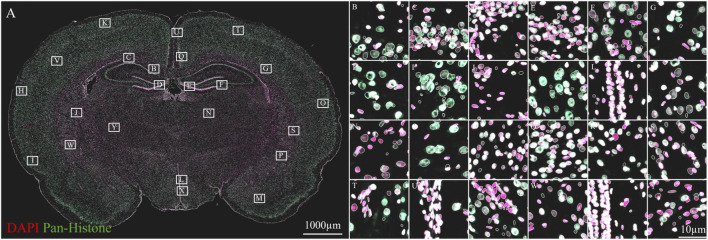
**(A)** Whole brain slice with labeled regions **(B–Y)** indicating selected locations. **(B–Y)** Close-up segmentation results cropped from the corresponding regions in **(A)**, demonstrating the proposed model’s ability to segment overlapping nuclei despite variability. The complete high-resolution segmentation files for slices S1–S4 are available in the electronic supplement.

### 4.1 Automated (unsupervised) segmentation quality assessment metrics

As noted earlier, our goal is to assess segmentation quality in a fully automated manner, without the need for human-generated ground truth annotations. We recognize that this goal cannot be met for instance segmentation in general. However, in the context of immunofluorescence imaging, especially multiplex imaging, it is indeed possible to develop practically useful quality metrics by examining the extent to which the fluorescence signal is accounted for by the segmentation masks (“signal coverage”), and the extent to which each object represents an individual cell of a certain type as indicated by the expression of a cell-type marker (we call that marker purity). These metrics are described below.

#### 4.1.1 Signal coverage metric Γ

This is a pixel-level measure that quantifies the number of foreground pixels that are missed by all the segmentation masks generated by the proposed model, collectively denoted $M_{model}$ . For this, we identify all the foreground pixels $M_{DAPI,Histone}$ by fitting a two-component Gaussian Mixture Model (GMM) to separate foreground and background. With this, the coverage metric $\Gamma \in \left(0,1\right)$ over the entire image is formulated as shown in Equation 8:

$$\Gamma =\frac{\sum_{\left(x,y\right)}\left(M_{model}\cap M_{DAPI,Histone}\right)\;}{\sum_{\left(x,y\right)}M_{DAPI,Histone}}$$ (8)

where $\left(x,y\right)$ denotes all the pixel locations.

#### 4.1.2 Marker purity metric Π

This measure analyzes the expression of cell-type protein markers over each object, as illustrated in Figure 6. We consider an object to be ‘pure’ if it contains foreground pixels for 1 cell type alone, and ‘impure’ otherwise. To make this notion precise, we model a nuclear mask $M_{n}$ as the union of multiple cell-type-specific masks $M_{n}^{c}$ , with weights $\alpha_{n}^{c}\in \left(0,1\right)$ , where c=1,..C indexes the cell-type marker channels, and $\alpha_{n}=\left[\alpha_{n}^{1},\ldots \alpha_{n}^{C}\right]$ is the vector of these weights. We perform a sparse decomposition using Orthogonal Matching Pursuit (OMP) (Pati et al., 1993) to find the most significant cell-type masks and use the corresponding coefficients $\alpha_{n}^{c}$ to define the purity metric for a single mask $M_{n}$ which can be obtained by solving the optimization problem in Equation 9.

$${\hat{\alpha }}_{n}=\underset{\alpha_{n}\;}{\mathrm{argmin}}{\left\|\text{ReLuOMP}\left(M_{n}-\sum_{c=1}^{C}\alpha_{n}^{c}M_{n}^{c}\right)\right\|}_{2}^{2},s.t.{\left\|\alpha_{n}\right\|}_{0}\leq k$$ (9)

where k is the $L_{0}$ sparsity constraint (we use $\left.k=3\right)$ , and ReLuOMP is the rectified linear unit function.

**FIGURE 6:**
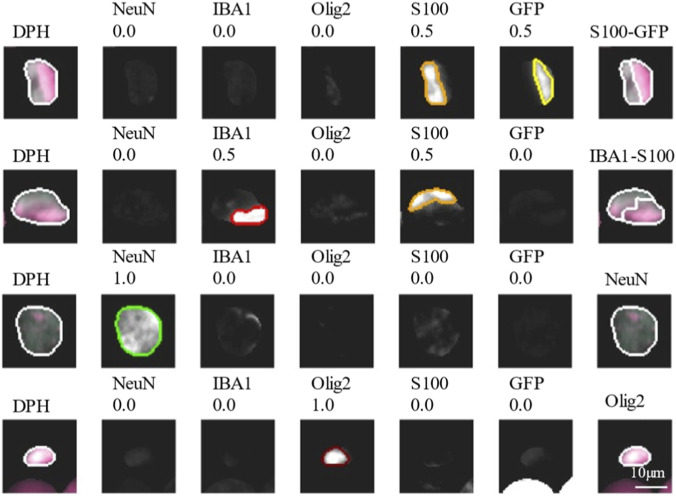
Four examples illustrate the sparse decomposition method for measuring the marker purity for cells. DPH represents DAPI and Pan-Histone. The first column shows the nuclear segmentations, and the second to sixth columns show the segmentations of the signals in five different channels containing cell-type markers. The last column shows the final type. The first row shows an example of cell with low purity (0.5) since it contains a mixture of S100 (0.5), and GFP (0.5) signals. The second row shows an example of cell with low purity (0.5) since it contains a mixture of IBA1 (0.5), and S100 (0.5) signals. The third and fourth row show high purity (1.0) examples.

With this, the purity metric for the $n^{th}$ object is given by $\Pi_{n}=\max\left(\alpha_{n}^{1},\ldots \alpha_{n}^{C}\right)$ , and the global purity metric $\Pi \in \left(0,1\right)$ over all N detected masks is given by Equation 10:

$$\Pi =\frac{1}{N}\sum_{n=1}^{N}\Pi_{n}$$ (10)

The object-wise purity metric $\Pi_{n}$ can also be used to identify erroneously segmented objects for corrective editing if desired. For the cell-type-specific masks $M_{n}^{c}$ , we use a GMM model to identify the foreground pixels in the cell-type marker channel. In our case, the biomarkers were NeuN for neurons, Iba-1 for microglia, Olig2 for oligodendrocytes, S100β for astrocytes and GFP for endothelial cells. Specifically, we first fit a GMM with three components and then identify the second largest and largest GMM clusters based on their mean intensity values. We then calculate the maximum intensity within the second-largest cluster and the minimum intensity within the largest cluster. The average of these two values is used as the foreground threshold. This method is simple yet effective, and filters out the cytoplasm, processes and auto-fluorescence signals.

While the proposed metrics, signal coverage and marker purity**,** provide biologically interpretable indicators of segmentation quality in multiplex imaging, these metrics are conceptually aligned with the design goals of our method. As a result, segmentation approaches that intentionally exclude dim or partial nuclei may obtain lower coverage scores despite representing reasonable design choices. Similarly, marker purity assumes largely coherent marker expression within nuclei and may penalize biologically valid transitional or mixed phenotypic states. Therefore, these metrics should be interpreted as complementary diagnostic indicators rather than absolute measures of segmentation correctness.

### 4.2 Performance summary

Tables 1, 2 provide a performance summary of the proposed *M* ^*3*^ *-RCNN* model against five benchmarks (*Cellpose2.0*, *StarDist*, *SAM-C*, *MRCNN*, *MRCNN-AUG*). For the *Cellpose2.0* and *StarDist* models, we used their default parameter settings. For the *SAM-C* method, we provided a 256 × 256 array of point prompts to *SAM* as input and used our rule-based filtering Supplementary Algorithm 1 to eliminate minor errors. For *MRCNN*, we trained the *MRCNN* model on annotations generated by *SAM*, which were filtered using Supplementary Algorithm 1. The *MRCNN-AUG* model was trained on the annotations generated by *SAM*, which were filtered using Supplementary Algorithm 1, and in addition, we applied data augmentation using Supplementary Algorithm 2.

The proposed (strong) model was trained using the same annotations as the *MRCNN-AUG* model. To ensure a fair comparison, we trained *M* ^*3*^ *-RCNN*, *MRCNN*, and *MRCNN-AUG* for the 5000 iterations with a batch size 32, $\lambda_{1}=\lambda_{2}=\lambda_{3}=1,\beta_{1}=0.8$ , $\beta_{2}=0.7$ , $\beta_{3}=0.5$ , t=3 , utilizing the same backbone ResNet-50, model hyperparameters, and post-processing. Post-processing was performed after supervised evaluation in two stages. First, false positive detections were removed by cross-referencing the results with those from *MRCNN*. Then, potentially missed detections were recovered by incorporating segmentation outputs from *Cellpose2.0* and *StarDist*. For fair comparison, we report Cellpose2.0, StarDist, and SAM-C using their native outputs (default settings), while the two-stage post-processing is applied consistently only to the Mask R-CNN–based pipeline variants (MRCNN, MRCNN-AUG, and M3-RCNN) as part of the proposed end-to-end workflow.

For the S1 dataset, our method achieves the highest AJI+ and PQ scores. *MRCNN* and *MRCNN-AUG* both outperform *Cellpose2.0*, *StarDist* and *SAM-C.* In terms of the purity and coverage metrics for the S1 - S4 datasets, all three methods including *M* ^*3*^ *-RCNN, MRCNN* and *MRCNN-AUG* outperform *Cellpose2.0*, *StarDist* and *SAM-C*. This trend aligns with the results obtained using supervised metrics, further validating the reliability of our proposed evaluation metric. Importantly, our conclusions regarding segmentation quality are not based solely on these unsupervised metrics but are also supported by supervised benchmarks (AJI+, PQ) and qualitative inspection. We can also see that our baseline *SAM-C* has surpassed *StarDist* and *Cellpose2.0* due to its training on a large dataset, giving it a better generalization ability. It is not surprising that *Cellpose2.0* and *StarDist* do not perform as well since they have not been trained on this specific dataset. *M* ^*3*^ *-RCNN*, *MRCNN* and *MRCNN-AUG* outperform *SAM-C* which shows the beneficial effect of the coverage expansion phenomenon in weak to strong generalization.

We also examined the sensitivity of the pseudo-label generation process to the density of SAM point prompts. Specifically, we varied the points_per_side parameter of the SAM automatic mask generator and evaluated the resulting segmentation quality. The results are summarized in Table 3. Sparse prompting (32) leads to insufficient spatial coverage and poor segmentation performance. Increasing the density to 64 substantially improves AJI+, PQ, and the unsupervised metrics (coverage and purity). Further increasing the density to 128 yields only marginal improvements, indicating diminishing returns once sufficient spatial coverage is achieved.

**TABLE 3:** Summary of results from the ablation study.

Points per side	AJI+	PQ
32	17.0%	17.0%
64	77.3%	74.0%
128	76.0%	77.0%

Table 4 provides an ablation study of the proposed *M* ^*3*^ *-RCNN* model on key parameters and components. Removing ECA module or multi-heads causes a decrease in performance, showing that each component contributes independently. The complete model which combines both modules achieves the best performance. As shown in Figure 5, our model effectively segments entire nuclei for overlapping cases, even in the densely packed regions.

**TABLE 4:** Prompt-density sensitivity analysis for SAM pseudo-label generation.

Components		S1	
ECA	Multi-heads	AJI+	PQ
✓		77.0%	76.3%
	✓	77.1%	76.5%
✓	✓	**77.3%**	**76.7%**

### 4.3 Evidence for weak to strong generalization

*SAM-C* serves as the weak teacher, and the proposed *M* ^*3*^ *-RCNN* serves as the strong student model in the weak to strong generalization framework. The data augmentation in Supplementary Algorithm 2 is a crucial link between the weak teacher and the strong student model. As shown in Table 1, the *M* ^*3*^ *-RCNN* strong student model outperforms the teacher model on the 100-tile ground-truth dataset that was not seen during training. This observation is consistent with the coverage expansion phenomenon described in weak-to-strong learning, in which the student model can generalize to instances where the teacher lacks confidence or to data points that are not part of the original training annotations.

Next, we compared the pseudo-label data before and after weak-to-strong learning to examine evidence for the pseudo-label correction phenomenon (a well-trained student model can learn to correct the weaker model’s mistakes) in feature space (Figure 7). The detailed feature extraction procedure and theoretical analysis are described in Supplementary Algorithm A4. In summary, our analyses suggest the presence of two phenomena commonly associated with weak-to-strong learning: pseudo-label correction and coverage expansion. These observations are consistent with the improved nuclear segmentation performance observed in our experiments. However, these observations should be interpreted as qualitative and theoretical indications rather than definitive validation, since weak-to-strong learning theory has primarily been developed for classification tasks and its extension to instance segmentation remains an open research problem.

**FIGURE 7:**
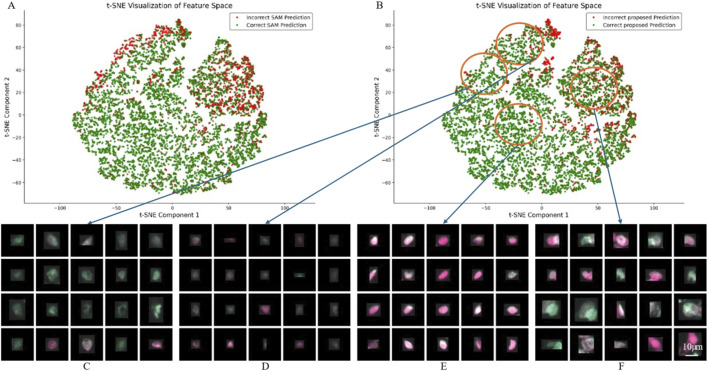
Illustration of pseudo-label correction in feature space. **(A)** t-SNE visualize nuclei in the pseudo-label dataset before weak-to-strong learning, where red and green indicate incorrect and correct predictions. **(B)** After weak-to-strong learning, more nuclei are correctly predicted. Representative clusters **(C–F)** highlight improved segmentation of dim and overlapping nuclei.

## 5 Conclusions and discussion

We have demonstrated the practicality of building strong (more capable) nuclear segmentation models from weak (less capable) models in a fully automated manner under the framework of weak to strong generalization, for which evidence is clearly observed. Importantly, the entire process, starting from the image to segmentation results accompanied by performance metrics, is fully automated and does not require any manual annotation effort. *This strategy is made possible by the availability of versatile and powerful foundational models like SAM, and the exploitation of image cues that are available in multiplex IF whole slide images*. Data cleanup and augmentation algorithms are the unsung but crucial enabler of weak to strong generalization.

Our method can be used to segment spatial proteomics images in a fully automated manner. The first run of the proposed method on a new dataset produces two outcomes: (i) accurate fully automated segmentation results for the new dataset accompanied by automated assessment metrics; and (ii) a trained *M* ^*3*^ *-RCNN* model that can be used for segmenting (in inference mode) a larger collection of similar datasets (e.g., images from the same instrument using the same protocols). For whole slide images (WSIs) with dimensions of 29,398 × 43,054 pixels, our pipeline is capable of generating complete image predictions within ∼9 h (1 min to divide WSIs into tiles, 4 h 25 min to generate masks using SAM, 11 min to run Supplementary Algorithm 1, 2 min to run Supplementary Algorithm 2, 2 h for training, 1 h 2 min for inferencing and postprocessing, 57 min for merging results) on a computer with six NVIDIA RTX6000A GPUs, fully automatically. Once trained, subsequent inferencing using the model takes ∼0.01 s per tile in the WSI image with 6,012 tiles (∼1 h total). This breakdown highlights that SAM pseudo-label generation is the dominant cost, so prompt density (and related SAM settings) primarily impacts runtime through the pseudo-label generation stage.

By introducing ECA module and multi mask heads, our *M* ^*3*^ *-RCNN* model learned capabilities that are not present in the Mask-RCNN model (e.g., improved handling of overlapped nuclei). The proposed automated (unsupervised) evaluation metrics offer the ability to assess automated segmentation quality without the need for human inspection and proofreading. This becomes more valuable in the current content when automated multiplex IF systems are routinely producing massive images on a sufficiently large scale that manual proofreading is unaffordable. When manual editing is needed, the purity metric $\Pi_{n}$ can be used to identify the subset of objects that need corrective editing (efficient analytics-driven proofreading).

The proposed model is amenable to further refinement and can be potentially adapted to other image analysis tasks. Spatial proteomics experiments often vary substantially in channel composition, staining targets, and data quality, depending on the biological question and experimental design. In this study, our model leverages eight multiplex IF channels that are closely associated with nuclear morphology and surrounding cellular context. The availability of these complementary signals provides richer information than single-channel or few-channel inputs, which helps improve segmentation robustness. Accordingly, the current work is primarily scoped to multiplex IF datasets with sufficiently informative multi-channel staining. In other tissues or imaging settings with different channel combinations, staining chemistries, or fewer available channels, the framework may require adaptation to account for the available signal characteristics. Going forward, it is possible to develop even stronger models and update the cleanup and augmentation modules in concert to develop novel capabilities, with full automation. Even with brain tissue images, there is an opportunity to refine the method in extremely dense regions (e.g., the hippocampus). While our data augmentation algorithm cannot fully simulate the full range of nuclear overlapping scenarios in such regions, we note that human analysis of these regions is so challenging that reliable human annotations are difficult to obtain. Ultimately, the proposed method cannot generalize to cases that are entirely absent from the training set.

## Data Availability

The original contributions presented in the study are included in the article/Supplementary Material, further inquiries can be directed to the corresponding author.
