# Supplementary material for: Weak-to-strong generalization enables fully automated training of multi-head mask-RCNN model for segmenting densely overlapping cell nuclei in multiplex whole-slice brain images
Source: Front Bioinform. 2026 May 11;6:1733655. doi: 10.3389/fbinf.2026.1733655 (PMC13198928; doi:10.3389/fbinf.2026.1733655)
Supplement: Supplementary file 1 [file Supplementaryfile1.docx]

**Appendix:**

**A1 - Algorithm 1: Rule-based filtering**

| **Input:**$D={\{(x_{i}^{DAPI,Histone},\varepsilon_{i})\}}_{i=1}^{K},$ $\varepsilon_{i}={\{e_{i,j}\}}_{j=1}^{N_{i}},$parameters $\beta_{1}$, $\beta_{2},\beta_{3}$  **Output:** $D^{filtered}={\{(x_{i}^{DAPI,Histone},\varepsilon_{i}^{filtered})\}}_{i=1}^{K}$, $\varepsilon_{i}^{filtered}={\{e_{i,j}^{filtered}\}}_{j=1}^{N_{i}^{filtered}}$ | |
| --- | --- |
| 1 | **for** $i$ = 0 **to** $K$ **do** |
| 2 | $\varepsilon_{i}^{u\_filtered}=\{ \}$ # Store non-union masks |
| 3 | **for** $j$ = 0 **to** $N_{i}$ **do** |
| 4 | $\varepsilon_{i}^{components}=\{ \}$ # Store components masks |
| 5 | **for** $m$ = 0 **to** $N_{i}$ **do** |
| 6 | **if** $j \neq m$ **and** $SumPixels\frac{\left( e_{i,j}\cap e_{i,m} \right)}{SumPixels(e_{i,m})}>\beta_{1}$, **then** add $e_{i,m}$ to $\varepsilon_{i}^{components}$ |
| 7 | **end** **for** |
| 8 | **if (**$\frac{SumPixels( merge(\varepsilon_{i}^{components})\cap e_{i,j} ))}{SumPixels\left( e_{i,j} \right)}>\beta_{2}$ **and** $count\left( \varepsilon_{i}^{components} \right)>1$) **then** pass |
| 9 | **else** add $e_{i,j}$ to $\varepsilon_{i}^{u\_filtered}$ |
| 10 | **if** $isnuclei(x_{i}^{DAPI,Histone}, e_{i,j}-e_{i,m})$ **then** add $e_{i,j}-e_{i,m}$ to $\varepsilon_{i}^{u\_filtered}$ |
| 11 | **end** **for** |
| 12 | $\varepsilon_{i}^{d\_filtered}=\{ \}$ # Initialize an empty set to store non duplicate masks |
| 13 | **for** $p$ = 0 **to** $count(\varepsilon_{i}^{u\_filtered})$**do** |
| 14 | **for** $q$ = 0 **to** $count(\varepsilon_{i}^{u\_filtered})$**do** |
| 15 | **if** $p \neq q$ **and** $\frac{SumPixels(\varepsilon_{i,p}^{u\_filtered}\cap\varepsilon_{i,q}^{u\_filtered})}{SumPixels(\varepsilon_{i,p}^{u\_filtered})}>\beta_{3}$  **and** $\frac{SumPixels(\varepsilon_{i,p}^{u\_filtered})}{SumPixels(\varepsilon_{i,q}^{u\_filtered})}<1$ **then** pass |
| 16 | **else** add $\varepsilon_{i,p}^{u\_filtered}$ to $\varepsilon_{i}^{d\_filtered}$ |
| 17 | **if** $isnuclei(x_{i}^{DAPI,Histone},\varepsilon_{i,p}^{u\_filtered}-\varepsilon_{i,q}^{u\_filtered})$ **then** add $\varepsilon_{i,p}^{u\_filtered}-\varepsilon_{i,q}^{u\_filtered}$to $\varepsilon_{i}^{d\_filtered}$ |
| 18 | **end for** |
| 19 | **end for** |
| 20 | **for** $r$ = 0 **to** $count(\varepsilon_{i}^{d\_filtered})$**do** |
| 21 | **if** $notdim(x_{i}^{DAPI,Histone},\varepsilon_{i,r}^{d\_filtered})$ **then add** $\varepsilon_{i,r}^{d\_filtered}$to $\varepsilon_{i}^{filtered}$ |
| 22 | **end for** |
| 23 | **end for** |

**A2 - Algorithm 2: Data augmentation**

| **Input:**$D^{f}={\{(x_{i},\varepsilon_{i}^{f})\}}_{i=1}^{K}, \varepsilon_{i}^{f}={\{e_{i,j}^{f}\}}_{j=1}^{N_{i}^{f}},$parameter$t$  **Output:** $D^{aug}={\{(x_{i}^{aug},\varepsilon_{i}^{aug})\}}_{i=1}^{K},$ $\varepsilon_{i}^{aug}={\{e_{i,j}^{aug}\}}_{j=1}^{N_{i}^{aug}}$ | |
| --- | --- |
| 1 | **for** $i$ = 0 **to** $K$ **do** |
| 2 | ${obj}_{i}^{copy}$ = $eligible_{copy}(x_{i}, \varepsilon_{i}^{f})$ |
| 3 | ${obj}_{i}^{paste}$ = $eligible_{paste}(x_{i}, \varepsilon_{i}^{f})$ |
| 4 | **for** $j$ = 0 **to** $count({obj}_{i}^{copy})$**do** |
| 5 | ${obj}_{i}^{trans}$ = $augmenation\_transform({obj}_{i}^{copy} )$ |
| 6 | $x_{i}, \varepsilon_{i}^{f}=copy\_paste({obj}_{i}^{trans}, {obj}_{i}^{paste}, t, x_{i}, \varepsilon_{i}^{f})$ |
| 7 | **end for** |
| 8 | $x_{i}^{aug},\varepsilon_{i}^{aug}=$ $x_{i}, \varepsilon_{i}^{f}$ |
| 9 | **end for** |

**A3 - Data Augmentation for Simulating Cell Overlaps**

To generate a set of training examples of cell overlaps, we use a straightforward “copy and paste” strategy. Within each image $x_{i},$we select two nuclei from$\varepsilon_{i}^{f}$, one to serve as the ‘copy’ nucleus, which undergoes a combination of spatial and intensity transformations, and the other as the ‘paste’ nucleus, onto which the first nucleus is overlayed. Given that $\varepsilon_{i}^{f}$may still include noisy labels, we select the ‘copy’ and ‘paste’ nuclei based on four criteria: (1) choosing nuclei that are spatially isolated from others; (2) avoiding nuclei close to the image boundaries; (3) excluding nuclei with concave mask contours; and (4) discarding dim nuclei. For the transformation of ‘copy’ nuclei, we apply random rotation and opacity adjustments to the selected nuclei to simulate expected overlap patterns. To prevent extreme overlaps, we implement a threshold on the overlap ratio. The output of these augmentations is denoted$D^{aug}={\{(x_{i}^{aug},\varepsilon_{i}^{aug})\}}_{i=1}^{K}$, where$\varepsilon_{i}^{aug}={\{e_{i,j}^{aug}\}}_{j=1}^{N_{i}^{aug}}$, $N_{i}^{aug}$represents the revised instance count in the $i^{th}$ image.

**A4 - Feature-Space Analysis and Theoretical Error Bound**

For this, we extracted all nuclei from 100 labeled images from the training set by locating their centers and zero padded to 80×80 – pixel image patches. Each such patch (DAPI and Pan-histone) is represented by a seven-dimensional (7-D) feature vector that quantifies segmentation difficulty in terms of: (i) foreground contrast; (ii) occlusion score; (iii) boundary variability; (iv) nucleus size; (v) aspect ratio; (vi) edge intensity; and (vii) background variability. Foreground contrast measures the difference in intensity between the nucleus and its background. Occlusion score evaluates the proportion of a nucleus that is overlapped by other nuclei. Boundary variability evaluates the number of edge pixels relative to nucleus size. Nucleus size normalizes the nucleus area by image size. Aspect Ratio is the width-to-height ratio. Edge intensity is the mean intensity of nucleus edges. Lastly, Background variability measures the standard deviation of background pixel intensities.

We used the t-SNE algorithm to project these 7-D features to the plane for visualization and examined close-ups of image regions for visual confirmation. To determine whether a prediction is correct (green) or incorrect (red), we used the following criteria. For non-overlapping nuclei, a prediction is considered correct if its IoU with the ground truth exceeds 0.7. For overlapping nuclei, a prediction must cover at least 10 pixels of the overlapping region and have an IoU > 0.5 to be considered as correct. The t-SNE projection is used only for visualization purposes. Correct and incorrect predictions are colored in green and red, respectively, based on the IoU criteria described above. Additionally, by visualizing the clusters of data points, particularly in regions where pseudo-label correction occurred, we see that our model achieves better segmentation of large, small and dim nuclei, as well as nuclei occluded by other nuclei. Although t-SNE is qualitative and parameter-sensitive, the clustering patterns are consistent with pseudo-label correction during weak-to-strong learning.

Lang *et al*. ([Lang et al., 2024](#_heading=h.4d9errn63nx5)) have calculated an upper bound for the error rate $err\left( S_{i} \right)$ of a student model against ground truth data on a pseudo labeled training subset $S_{i}$ with known ground truth. Their estimate was formulated for classification rather than segmentation problems. With this in mind, we choose to consider our model *M^3^-RCNN* as a classifier $f$ that classifies image patches, denoted $x,$ that are centered on nuclei. Following the notation of Lang *et al*., we denote $y$ as the ground truth labeler, and $\tilde{y}$ as the pseudo labeler *SAM-C.* With this notation,$err\left( S_{i} \right)$ is the error rate for $f$ against the ground truth labels, where $S_{i}$ represents the set of extracted image patches around the nuclei. We use $S_{i}^{bad}$to denote the incorrectly pseudo labeled image patches and $S_{i}^{good}$ to denote the correctly pseudo labeled patches. With this, the upper bound for $err\left( S_{i} \right)$ is given by **Equation S1**:

| $err\left( S_{i} \right)\leq\frac{2\alpha_{i}}{1-2\alpha_{i}}P\left( S_{i} \right)+err\left( S_{i} \right)+\alpha_{i}(1 - \frac{3}{2}c)$, | (S1) |
| --- | --- |

where $\alpha_{i}=P(S_{i}^{bad}|S_{i})$ is the error rate of the pseudo labeler against ground truth labels, and $P\left( S_{i} \right)$ is a measure of the classifier $f$’s robustness, where a lower value corresponds to a higher robustness, and *vice versa*. Here, $R\left( f \right)=\{x:r\left( f,x \right)=0\},$ where $r\left( f,x \right)=P\left( x^{'}\in N\left( x \right) \right)$ is the probability that $f$ assigns different labels to $x$ and its neighbor $x^{'}$ in feature space. In **Equation S1**, $c$ is the expansion rate between $S_{i}^{bad}$ and $S_{i}^{good}$, which measures how well two sets are mixed together, and is calculated using the same formulation described in Lang *et al*. ([Lang et al., 2024](#_heading=h.4d9errn63nx5)). The calculated values are shown in **Supplementary Table S1**.

**Supplementary Table S1: Measured Expansion and Error Bounds**

| Model | $P(\left( S_{i} \right))$ | $c$ | $\alpha_{i}$ | $err(\left( S_{i} \right))$ | Bound | $err(\left( S_{i} \right))$ |
| --- | --- | --- | --- | --- | --- | --- |
| *M3-RCNN* | 0.15 | 0.43 | 0.19 | 0.17 | 0.32 | 0.16 |

Our model error rate $err\left( S_{i} \right)$ is smaller than $\alpha_{i}$, this indicates the occurrence of the pseudo correction phenomenon. Also, the upper bound of $err\left( S_{i} \right)$ equals 0.32, which is reasonably close to the actual observed value of 0.17, indicating that the theoretical estimation aligns well with empirical results.

**A5 - Supplemental Files**

The code, four sample WSI images (S1 – S4), and full-resolution segmentation results are provided in open form for viewing, community adoption, and potential adaptation to other biomedical image analysis tasks. The WSI images (S1 – S4) can be found at [figshare](https://doi.org/10.6084/m9.figshare.13731585.v1). The code and results are shared in [Dropbox](https://www.dropbox.com/scl/fo/utd5oxrn83f3qybf3l59y/AFF5w8N-QqiaVGSDOEFfFFg?rlkey=pilymavrojooqyuz23tg5xppj&st=gk6bn24v&dl=0).
